# An unusual triad: tuberculomas, tubercular meningitis, and hydrosyringomyelia in a child from a suburban region

**DOI:** 10.1186/s12245-025-01025-9

**Published:** 2025-10-24

**Authors:** Anand Hatgaonkar, Kajal Hatgaonkar

**Affiliations:** 1 Department of Radiodiagnosis, Datta Meghe Medical College, DMIHER (DU), Nagpur, Maharashtra India; 2 Department of Pathology, Datta Meghe Medical College, DMIHER (DU), Nagpur, Maharashtra India

**Keywords:** Tuberculomas, Tubercular meningitis, Hydrosyringomyelia, Magnetic resonance imaging, Suburban region

## Abstract

Central nervous system (CNS) tuberculosis is a rare but serious manifestation of Mycobacterium tuberculosis infection, especially in the paediatric population.

We report a rare case of an eight year old male child from a suburban region presenting with intracranial tuberculomas, tubercular meningitis, and spinal hydrosyringomyelia (HSM). Magnetic Resonance Imaging (MRI) revealed multiple brain tuberculomas, basal exudates, abnormal meningeal enhancement, spinal HSM with features of arachnoiditis. The patient was operated for removal of tuberculomas and placement of ventriculo-peritoneal (V-P) shunt and responded well to antitubercular therapy and corticosteroids.

Tuberculosis should be considered in the differential diagnosis in children presenting with unexplained neurological symptoms in endemic regions. Early neuroimaging is critical in avoiding morbidity and permanent neurological damage.

## Background

Tuberculosis (TB) remains a significant health challenge in India, with central nervous system (CNS) TB accounting for approximately 1% of all TB cases and up to 10% of extrapulmonary cases [1]. Intracranial tuberculomas and tuberculous meningitis (TBM) are well-described manifestations; however, hydrosyringomyelia (HSM) is a rare and underreported complication, especially in paediatric patients [2]. This case highlights the presence of spinal HSM as a complication of CNS TB in a child, emphasizing the importance of early detection and management.

## Case presentation

An eight year old male child from a suburban region presented with generalised tonic-clonic convulsions and weakness in both lower limbs for two months, and intermittent low-grade fever and weight loss over the last six months. Neurological assessment revealed spastic paraparesis and reduced pain and temperature sensation below the C3 dermatome. No cranial nerve deficits were observed.

**Fig. 1:**
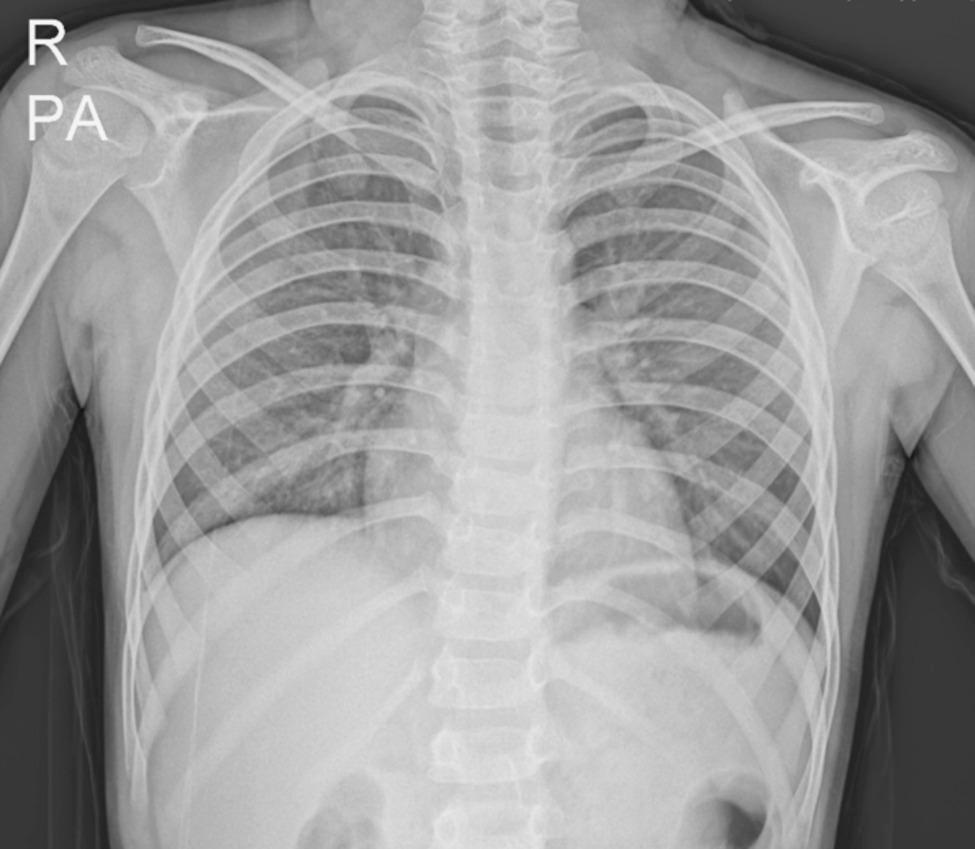
X-ray chest PA view. No significant abnormality detected

Chest X-ray revealed no significant abnormality (Fig. 1).

**Fig. 2:**
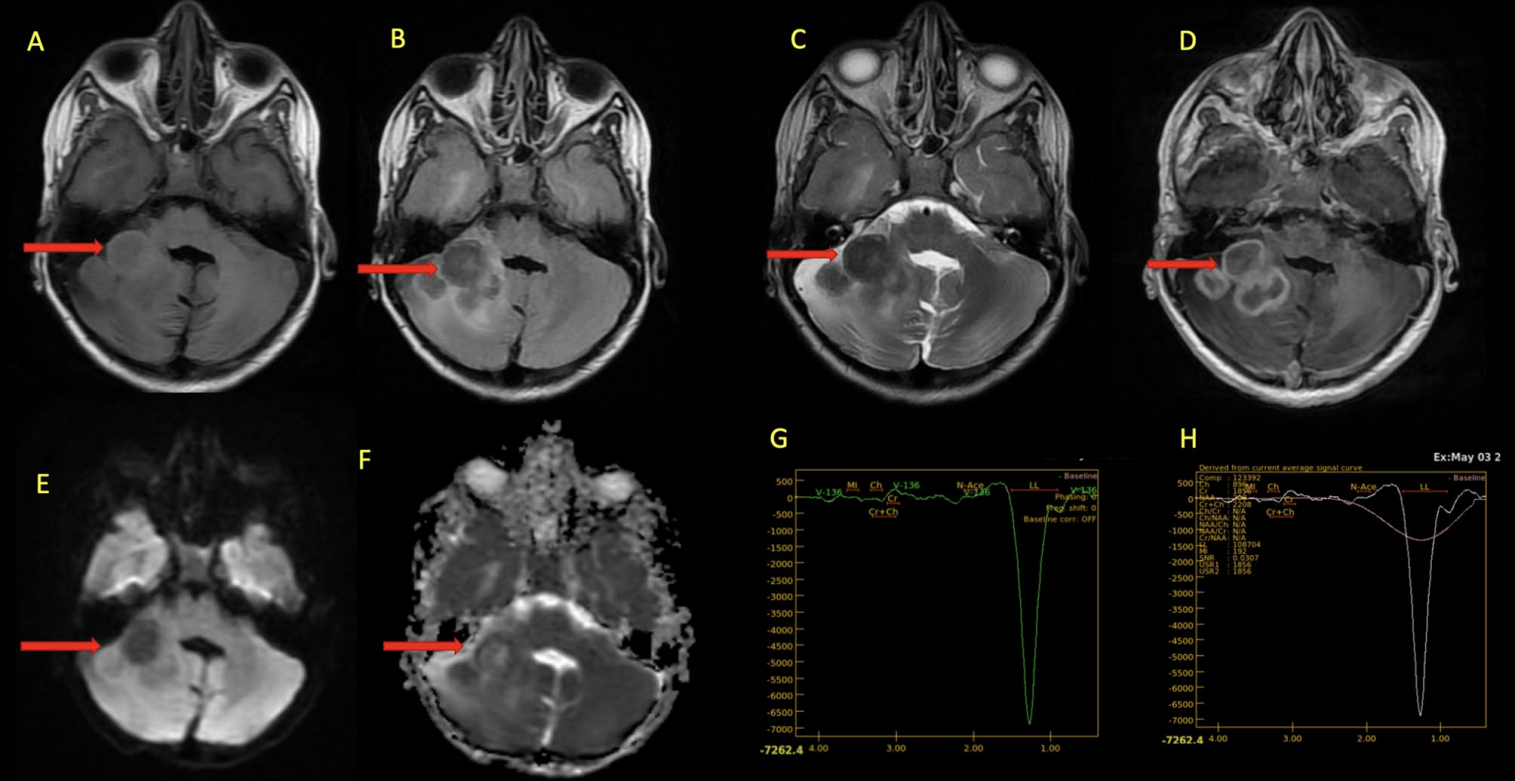
MRI brain. Axial images in T1W (**A**), FLAIR (**B**), T2W (**C**), post contrast T1 fat suppressed (**D**), DWI (**E**) and ADC (**F**) sequences showing tuberculomas in right cerebellar region (red arrow) appearing iso-hypointense on T1, T2 and FLAIR images; with postcontrast peripheral rim enhancement, no restricted diffusion with mild surrounding oedema and mass effect mildly compressing fourth ventricle and obliterating right CP angle cistern. MRS images (**G** & **H**) revealed high lipid lactate peak, reduced or Absent NAA, Choline, and Creatine

Magnetic Resonance Imaging (MRI) Brain revealed multiple ring-enhancing conglomerating lesions in right cerebellar region extending to right cerebello-pontine (CP) angle cistern appearing iso-hypointense on T1, T2 and FLAIR images with associated mild surrounding oedema and mass effect. Magnetic Resonance Spectroscopy (MRS) revealed high lipid lactate peak, reduced N-acetylaspartate (NAA), choline, and creatine. These imaging findings were consistent with tuberculomas (Fig. 2).

**Fig. 3:**
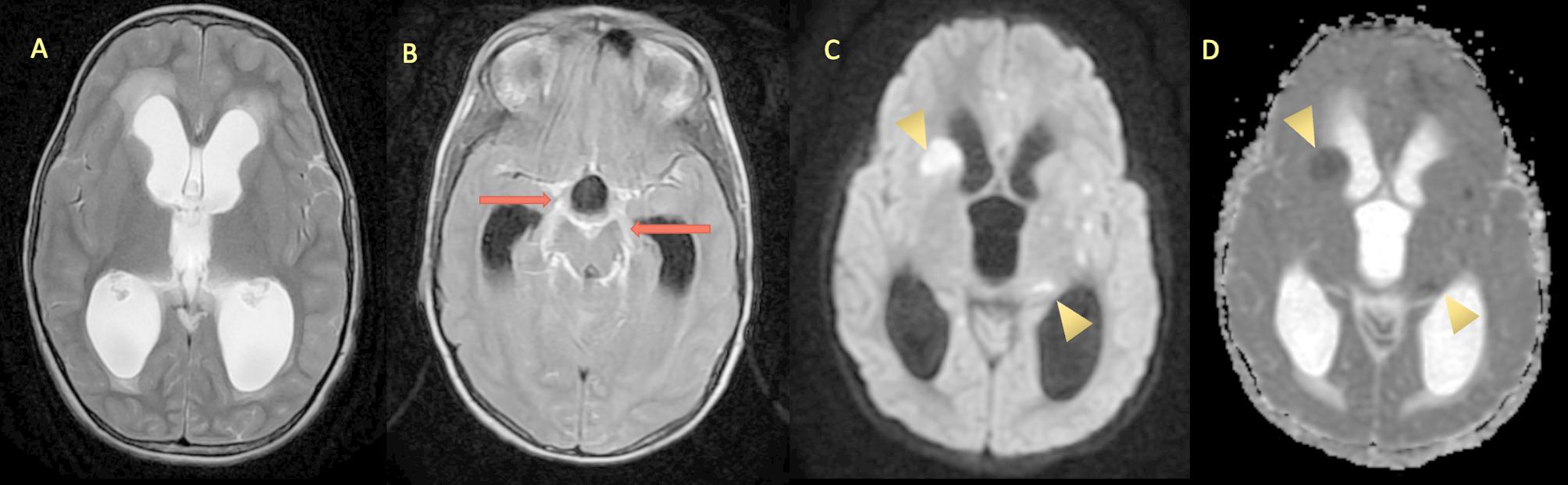
MRI brain. Axial T2W image (**A**) showing moderate hydrocephalus, post contrast T2 FLAIR axial image (**B**) demonstrating basal exudates with abnormal enhancement along the perimesencephalic and suprasellar cisterns (red arrow). Axial DW & ADC images (**C** & **D**) showing restriction of diffusion in the right caudate nucleus, and bilateral thalamostriate regions (yellow arrow heads)

Abnormal meningeal enhancement and exudates in basal cisterns suggested TBM. Moderate obstructive hydrocephalus with multiple ill-defined areas of diffusion restriction were noted involving right striato-capsular region, left thalamo-striato-capsular region, bilateral temporal lobes suggested acute ischemic insult (Fig. 3).

**Fig. 4:**
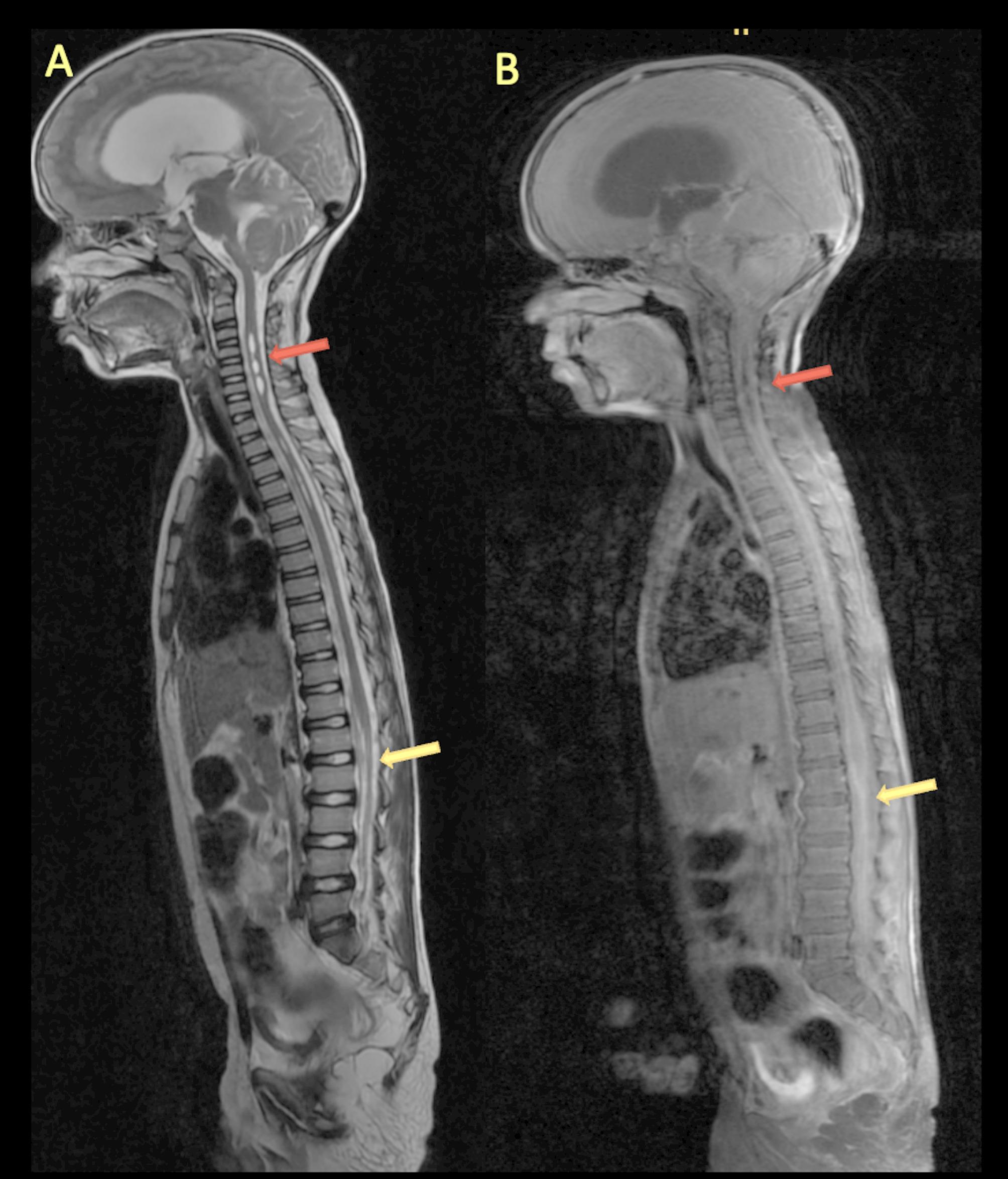
MRI spine. Sagittal T2 weighted image (**A**) showing dilatation of the central canal (hydrosyringomyelia) in cervical and thoracic cord (red arrow) and post contrast T1FS image (**B**) demonstrating extensive thickening and enhancement of the arachnoid, disappeared subarachnoid space, and nodular enhancement of conus medullaris (yellow arrow)

MRI Spine revealed focal dilatation of the central canal of the spinal cord suggesting HSM in cervico-thoracic cord extending from C3–C7 and D2-–D5 levels; clumping of nerve roots with dural and arachnoid enhancement suggestive of spinal arachnoiditis (Fig. 4).

On blood examination ESR was 100 mm/hr and blood counts showed lymphocytic leucocytosis. CSF analysis revealed; Protein: 130 mg/dL, Glucose: 30 mg/dL (blood glucose: 97 mg/dL), Cells: 85 cells/mm³ (90% lymphocytes), Ziehl Neelsen stain for Acid Fast Bacilli (AFB): Negative (Table 1).

**Table 1:** Laboratory investigations

Test Name	Patient Result	Normal Range
Blood Tests		
1. ESR	100 mm/hr	< 15 mm/hr
2. CBC- Total Leukocyte count	13,450 per mm^3^	4500–11,000 per mm^3^
3. CBC- Neutrophil	5776 per mm3	2500–8000 per mm^3^
4. CBC- Lymphocyte	6848 per mm3	1000–4000 per mm^3^
5. CBC- Eosinophil	540 per mm3	100–700 per mm^3^
6. CBC- Monocyte	258 per mm3	50–500 per mm^3^
7. CBC- Basophil	28 per mm3	25–100 per mm^3^
CSF Examination		
1. Protein:	130 mg/dL	18–48 mg/dL
2. Glucose	30 mg/dL	50–80 mg/dL
3. Microscopy- Cellularity	85 cells/mm³, 90% lymphocytes	< 5 cells/mm³, only lymphocytes
4. Ziehl Neelsen stain for Acid Fast Bacilli	Negative	Negative

*ESR* Erythrocyte Sedimentation Rate, *CBC *Complete Blood Count, *CSF *Cerebro Spinal Fluid

**Fig. 5:**
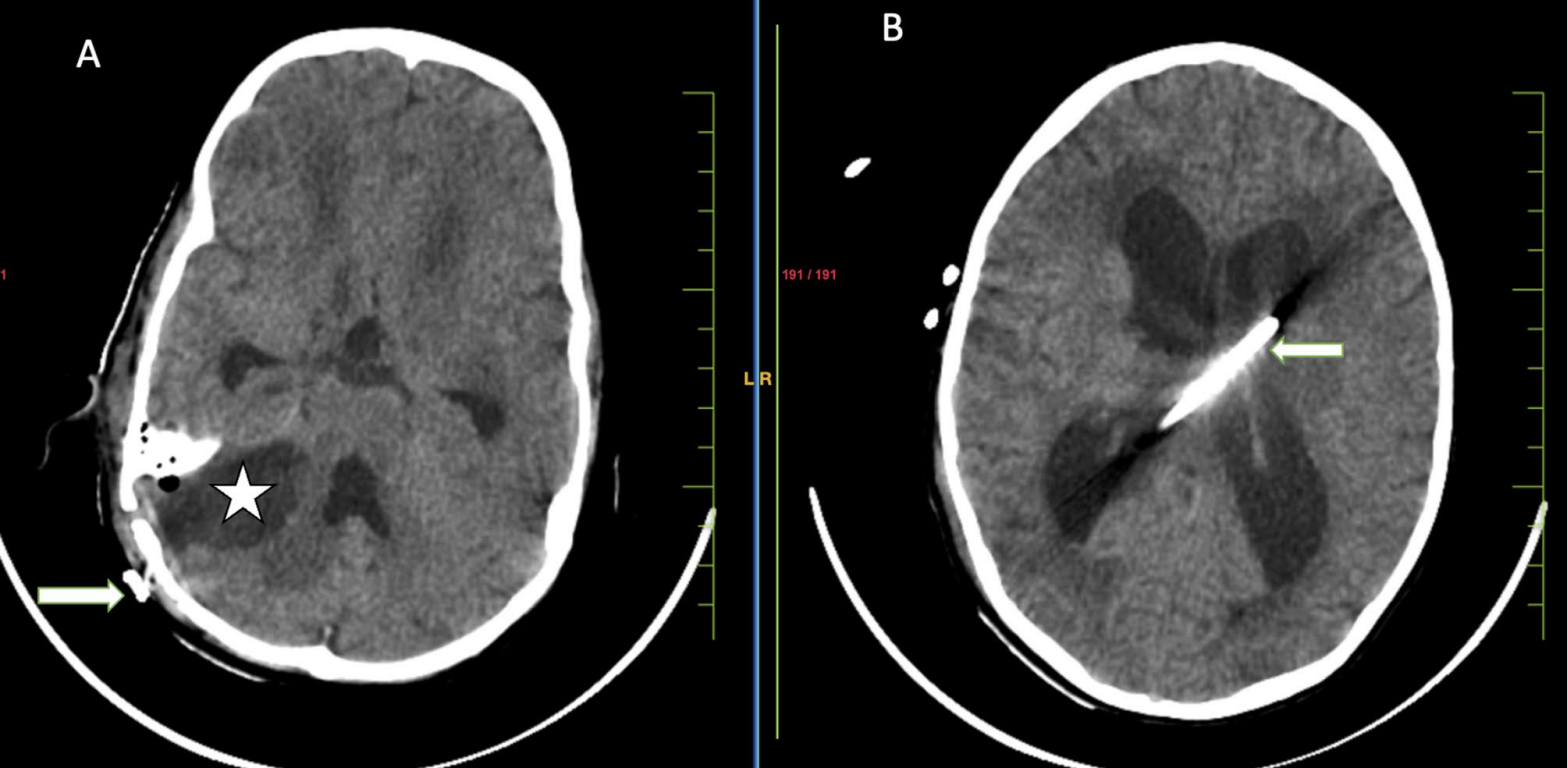
Post-operative non-contrast computed tomography (NCCT) brain. Axial CT images (**A** & **B**) showing right retromastoid suboccipital (RMSO) craniotomy defect with postoperative collection due to excised right cerebellar mass (★) and ventriculoperitoneal shunt (arrow)

Neurosurgical consultation was sought in view of deteriorating condition of the patient. Right retromastoid suboccipital (RMSO) craniotomy was done and tuberculomas were removed and ventriculo-peritoneal (V-P) medium pressure shunt was placed in view of obstructive hydrocephalous and raised intracranial pressure (Fig. 5).

**Fig. 6:**
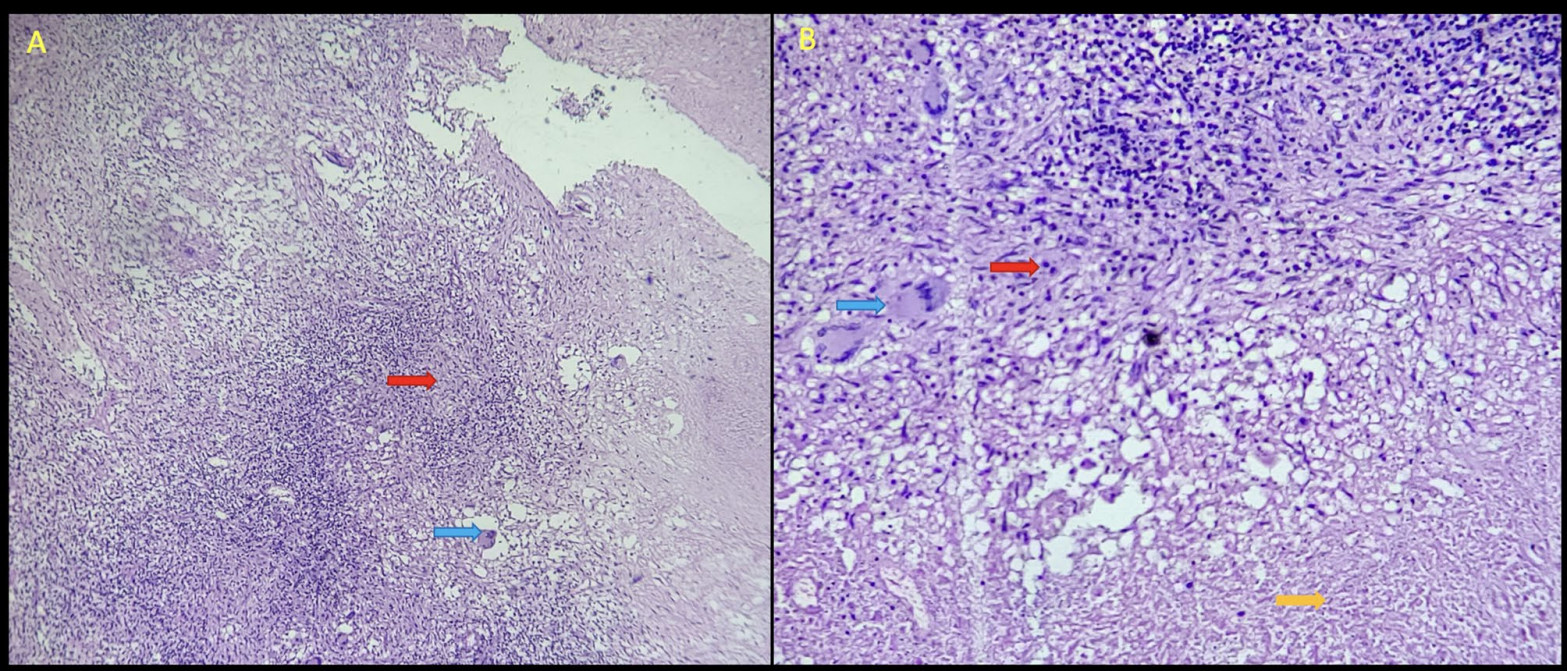
Histopathology. **A** Scanner view of the cerebellar lesion shows ill-formed epithelioid granulomas (red arrow) and multinucleated giant cells (blue arrow) with adjacent brain parenchyma (right side). 40x, Hematoxyline & Eosin stain. **B** Low power view shows caseating necrosis (yellow arrow), epithelioid cells (red arrow) and multinucleated giant cells (blue arrow). 100x, Hematoxyline & Eosin stain

Histopathological examination of the tissue removed from the CP angle revealed extensive areas of caseous necrosis and many well-formed granulomas composed of epithelioid cells, Langhans and Foreign body type multi- nucleated giant cells, surrounded by lymphocytes and fibrous tissue (Fig. 6).

The child was started on a standard 4-drug antitubercular treatment (ATT) containing isoniazid, rifampicin, pyrazinamide, and ethambutol for two months, to be followed by three drugs in the continuation phase with isoniazid, rifampicin, and ethambutol for another 10 months. He was also started on anti-seizure medication with valproic acid. Physiotherapy was recommended to address spasticity and motor recovery.

The child had shown significant improvement in neurological recovery during the admission period on ATT and steroids with no adverse effect of medication. The child was discharged after 15 days of admission and was advised to follow.

## Discussion

In paediatric patients the most likely source of CNS TB is from the pulmonary focus with hematogenous spread to the CNS. The meninges, the brain’s subpial or subependymal surfaces may be the site of the original Rich focus, which could lie dormant for many years. The precise stimulus required for the rupture and proliferation of these lesions is still not fully understood, however may be immunological in nature, depending upon the virulence of the bacteria and the immune resistance of the host [3, 4].

CNS TB can present as tubercular meningitis (TBM), focal or diffuse pachymeningitis, intracranial tuberculoma or brain abscess. Spinal TB can present as spinal meningitis and spinal arachnoiditis [3–6]. Spinal HSM is a rare complication of CNS TB. It is presumed to result from arachnoiditis-induced obstruction of CSF flow, leading to syrinx formation [6, 7]. Syrinxes are fluid-filled pockets that develop in the spinal cord, and this condition is called syringomyelia. Hydromyelia is described as the dilation of the central canal of the spinal cord. These two conditions are also known as HSM as they cannot be distinguished separately [8].

Neurological symptoms might range from agitation, lethargy to coma, depending on the stage of presentation. Patients with tuberculoma or tuberculous brain abscess may present with headache, convulsions, papilledema, or other symptoms of elevated intracranial pressure(3,4). In a spinal tuberculosis complicated by HSM, neurological symptoms can range in severity from minor discomfort to neurological impairments including sensory neural deficit and motor involvement [7].

Based on distinctive imaging and spectroscopic results, MRI can confidently diagnose tuberculomas. On T2W imaging, intracranial tuberculomas typically exhibit central hyperintensity or hypointensity with a hypointense rim, while on T1W images, they typically exhibit isointensity or hypointensity. Depending on central caseation, these granulomas exhibit either peripheral rim enhancement or homogeneous enhancement on fat suppressed T1W imaging following paramagnetic contrast injection. In vivo MRS can reveal decreased or absent NAA, choline, and creatine, as well as prominent lipid and lactate peaks [5, 9]. In TBM, imaging frequently reveals hydrocephalus, abnormal enhancement in the basal cisterns and acute ischaemic insult as a result of vasculitis [5, 9].

MRI characteristics of spinal meningitis and spinal arachnoiditis include matting of the nerve roots in the lumbar area, CSF loculation, and obliteration of the spinal subarachnoid space, together with a thinning of the spinal cord in the cervicothoracic spine. Arachnoiditis complications can include spinal cord involvement in the form of HSM and infarction [5, 7].

Based on the MRI findings and MRS results in the particular clinical scenario, which were backed by CSF results, we were able to confidently diagnosis tuberculomas, TBM and HSM in our case.

Although coexisting TBM and intramedullary or intracranial tuberculomas are reported in literature however a combination of tuberculomas, TBM, and Hydrosyringomyelia is a rare and very few cases are reported [7].

For children with severe ventriculomegaly, CSF diversion treatments must be carried out at the right time, particularly when patient has infracts as a complication, as in our patient. As far as tuberculomas in children are concerned, surgical intervention can be sought in patient with significant mass effect and in case with diagnostic dilemma to get histopathological insight. The need for surgical intervention has drastically reduced because of availability of effective antitubercular therapy (ATT); although multidrug resistance is a known challenge. Most lesions completely disappear with standard antitubercular treatment with measures to reduce cerebral oedema and mass effect using dexamethasone [10]. For HSM conservative treatment and high dose steroids suffice, use of intravenous immunoglobulin (IVIG) as part of the drug therapy with satisfactory outcome is documented [2].

##  Conclusion

CNS tuberculosis should be a differential diagnosis in children presenting with unexplained neurological symptoms in endemic regions. Hydrosyringomyelia, though rare, can occur due to spinal arachnoiditis. Early diagnosis with neuroimaging and prompt initiation of antitubercular treatment and corticosteroids can lead to good neurological recovery avoiding morbidity and permanent neurological damage.

## Data Availability

Not applicable.
